# Redermination of 9,9′-bianthracene-10,10′(9*H*,9′*H*)-dione

**DOI:** 10.1107/S1600536808028833

**Published:** 2008-09-13

**Authors:** Zhi-Gang Wen, Jia-Ming Li

**Affiliations:** a Department of Chemistry and Chemical Engineering, Qiannan Normal College for Nationalities, Duyun, Guizhou 558000, People’s Republic of China; b Department of Chemistry and Biology, Qinzhou University, Qinzhou, Guangxi 535000, People’s Republic of China

## Abstract

The crystal structure of the title compound, C_28_H_18_O_2_, was originally determined by Ehrenberg [(1967 ▶). *Acta Cryst*. **22**, 482–487] using intensity data obtained from Weissenberg photographs. The current determination provides a crystal and mol­ecular structure with a significantly higher precision and presents standard uncertainties on geometric parameters which are not available from the original work. The mol­ecule lies on a crystallographic twofold rotation axis which bis­ects the C—C bond [1.603 (3) Å] which joins the two anthracen-9(10*H*)-one units.

## Related literature

For general background, see: Li *et al.* (2002 ▶); Shi *et al.* (2004 ▶); Müller *et al.* (1996 ▶, 1998 ▶, 2001 ▶); Prinz, Burgemeister & Wiegrebe (1996 ▶); Prinz, Wiegrebe & Müller (1996 ▶). For related structures, see: Ehrenberg (1967 ▶).
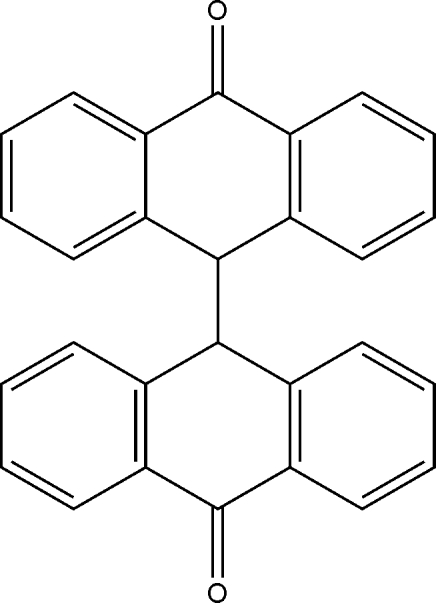


## Experimental

### 

#### Crystal data

C_28_H_18_O_2_
*M* *_r_* = 386.42
Monoclinic, 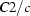

*a* = 22.295 (4) Å
*b* = 7.7297 (12) Å
*c* = 13.643 (2) Å
β = 126.768 (2)°
*V* = 1883.4 (5) Å^3^
*Z* = 4
Mo *K*α radiation
μ = 0.09 mm^−1^
*T* = 273 (2) K
0.22 × 0.18 × 0.15 mm

#### Data collection

Bruker SMART CCD diffractometer
Absorption correction: multi-scan (*SADABS*; Sheldrick, 1996 ▶) *T*_min_ = 0.982, *T*_max_ = 0.987
4785 measured reflections
1669 independent reflections
1172 reflections with *I* > 2σ(*I*)
*R*_int_ = 0.025

#### Refinement

*R*[*F* ^2^ > 2σ(*F* ^2^)] = 0.040
*wR*(*F* ^2^) = 0.105
*S* = 1.03
1669 reflections
136 parameters
H-atom parameters constrained
Δρ_max_ = 0.12 e Å^−3^
Δρ_min_ = −0.17 e Å^−3^

### 

Data collection: *SMART* (Bruker, 2003 ▶); cell refinement: *SAINT* (Bruker, 2003 ▶); data reduction: *SAINT*; program(s) used to solve structure: *SHELXS97* (Sheldrick, 2008 ▶); program(s) used to refine structure: *SHELXL97* (Sheldrick, 2008 ▶); molecular graphics: *SHELXTL* (Sheldrick, 2008 ▶); software used to prepare material for publication: *SHELXTL*.

## Supplementary Material

Crystal structure: contains datablocks global, I. DOI: 10.1107/S1600536808028833/lh2689sup1.cif

Structure factors: contains datablocks I. DOI: 10.1107/S1600536808028833/lh2689Isup2.hkl

Additional supplementary materials:  crystallographic information; 3D view; checkCIF report

## Figures and Tables

**Fig. 1.**
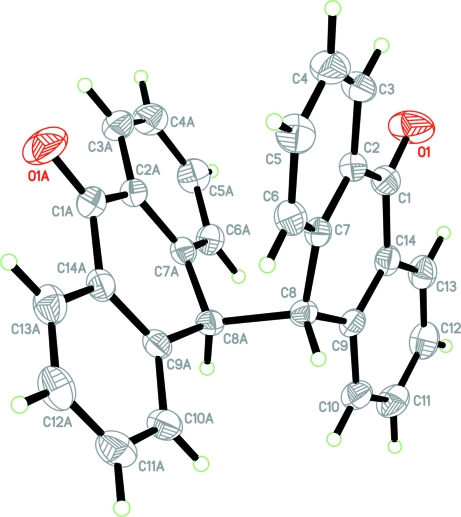
The molecular structure with displacement ellipsoids at the 30% probability level [symmetry code: (A) -x+2, y, -z+1/2].

## supplementary crystallographic information

### Comment

Synthesis of anthracenone derivatives have attracted great interest due to their interesting biological activities (Müller *et al.*, 1996, 1998, 2001; Prinz, Burgemeister & Wiegrebe, 1996; Prinz, Wiegrebe & Müller, 1996). Herein, we present a redetermination of the crystal structure of the title compound (I) which was originally refined in the non-conventional space group setting I2/a with unit cell parameters; a = 13.68 (4), b = 7.751 (3), c = 17.92 (4), β = 91.1 (3) (Ehrenberg, 1967). The current structure is of significantly higher precision than the orginal determination which was refined using intensity data obtained from Weissenberg photographs. The molecular structure of (I) is shown in Fig. 1. The molecule consists of two anthracen-9(10*H*)-one moieties linked together by a C—C [1.603 (3) Å] bond. A crystallographic twofold rotation axis bisects this bond.

### Experimental

Reagents and solvents used were of commercially available quality. The title complex (I) was synthesized according to the method of Shi *et al.* (2004) and Li *et al.* (2002). CF_3_COOH (40 ml) was added dropwise with stirring to a solution of anthracene-9,10-dione (5.0 mmol) in 15 ml of anhydrous CH_2_Cl_2_. The mixture was then placed in an ice bath and NaBH_4_ (0.95 g, 25 mmol) was added in portions. The resulting mixture was stirred for 24 h at room temperature. The reaction mixture was poured into 200 ml ice-water. The organic layer was extracted with CH_2_Cl_2_, dried over Na_2_SO_4_ and evaporated *in vacuo*. The crude product was recrystallized from toluene twice to give the main product 9,9'-bianthracene-10,10'(9*H*,9'H)-dione.

### Refinement

H atoms were positioned geometrically and refined using a riding model, with C—H = 0.93–0.98 Å and with *U*_iso_(H) = 1.2 times *U*_eq_(C).

### Crystal data

**Table:**

C_28_H_18_O_2_	*F*(000) = 808
*M**_r_* = 386.42	*D*_x_ = 1.363 Mg m^−^^3^
Monoclinic, *C*2/*c*	Mo *K*α radiation, λ = 0.71073 Å
Hall symbol: -C 2yc	Cell parameters from 1069 reflections
*a* = 22.295 (4) Å	θ = 2.9–24.7°
*b* = 7.7297 (12) Å	µ = 0.09 mm^−^^1^
*c* = 13.643 (2) Å	*T* = 273 K
β = 126.768 (2)°	Block, yellow
*V* = 1883.4 (5) Å^3^	0.22 × 0.18 × 0.15 mm
*Z* = 4	

### Data collection

**Table:**

Bruker SMART CCD diffractometer	1669 independent reflections
Radiation source: fine-focus sealed tube	1172 reflections with *I* > 2σ(*I*)
graphite	*R*_int_ = 0.025
φ and ω scans	θ_max_ = 25.1°, θ_min_ = 2.3°
Absorption correction: multi-scan (SADABS; Sheldrick, 1996)	*h* = −20→26
*T*_min_ = 0.982, *T*_max_ = 0.987	*k* = −9→9
4785 measured reflections	*l* = −16→8

### Refinement

**Table:**

Refinement on *F*^2^	Primary atom site location: structure-invariant direct methods
Least-squares matrix: full	Secondary atom site location: difference Fourier map
*R*[*F*^2^ > 2σ(*F*^2^)] = 0.040	Hydrogen site location: inferred from neighbouring sites
*wR*(*F*^2^) = 0.106	H-atom parameters constrained
*S* = 1.03	*w* = 1/[σ^2^(*F*_o_^2^) + (0.0478*P*)^2^ + 0.4019*P*] where *P* = (*F*_o_^2^ + 2*F*_c_^2^)/3
1669 reflections	(Δ/σ)_max_ = 0.001
136 parameters	Δρ_max_ = 0.12 e Å^−^^3^
0 restraints	Δρ_min_ = −0.17 e Å^−^^3^

### Special details

**Table:**

Geometry. All e.s.d.'s (except the e.s.d. in the dihedral angle between two l.s. planes) are estimated using the full covariance matrix. The cell e.s.d.'s are taken into account individually in the estimation of e.s.d.'s in distances, angles and torsion angles; correlations between e.s.d.'s in cell parameters are only used when they are defined by crystal symmetry. An approximate (isotropic) treatment of cell e.s.d.'s is used for estimating e.s.d.'s involving l.s. planes.
Refinement. Refinement of *F*^2^ against ALL reflections. The weighted *R*-factor *wR* and goodness of fit *S* are based on *F*^2^, conventional *R*-factors *R* are based on *F*, with *F* set to zero for negative *F*^2^. The threshold expression of *F*^2^ > σ(*F*^2^) is used only for calculating *R*-factors(gt) *etc*. and is not relevant to the choice of reflections for refinement. *R*-factors based on *F*^2^ are statistically about twice as large as those based on *F*, and *R*- factors based on ALL data will be even larger.

### Fractional atomic coordinates and isotropic or equivalent isotropic displacement parameters (Å^2^)

**Table:**

	*x*	*y*	*z*	*U*_iso_*/*U*_eq_	
O1	0.86707 (8)	−0.25682 (16)	0.29866 (15)	0.0855 (5)	
C1	0.90675 (10)	−0.1382 (2)	0.30813 (16)	0.0503 (4)	
C2	0.98793 (9)	−0.16248 (19)	0.37296 (14)	0.0423 (4)	
C3	1.02019 (10)	−0.3196 (2)	0.43139 (15)	0.0546 (5)	
H3	0.9900	−0.4083	0.4254	0.066*	
C4	1.09595 (11)	−0.3451 (2)	0.49762 (16)	0.0616 (5)	
H4	1.1169	−0.4513	0.5346	0.074*	
C5	1.14056 (10)	−0.2121 (2)	0.50886 (15)	0.0572 (5)	
H5	1.1921	−0.2277	0.5553	0.069*	
C6	1.10969 (8)	−0.0560 (2)	0.45207 (13)	0.0457 (4)	
H6	1.1408	0.0332	0.4616	0.055*	
C7	1.03286 (8)	−0.02957 (19)	0.38070 (13)	0.0379 (4)	
C8	0.99831 (8)	0.13334 (18)	0.30704 (13)	0.0370 (4)	
H8	1.0288	0.2306	0.3599	0.044*	
C9	0.91944 (8)	0.16565 (19)	0.26300 (13)	0.0393 (4)	
C10	0.88717 (9)	0.3276 (2)	0.21705 (15)	0.0491 (4)	
H10	0.9158	0.4170	0.2191	0.059*	
C11	0.81329 (10)	0.3570 (2)	0.16860 (16)	0.0591 (5)	
H11	0.7926	0.4660	0.1382	0.071*	
C12	0.76987 (10)	0.2268 (3)	0.16480 (16)	0.0589 (5)	
H12	0.7199	0.2471	0.1314	0.071*	
C13	0.80076 (9)	0.0673 (2)	0.21051 (15)	0.0535 (5)	
H13	0.7716	−0.0207	0.2086	0.064*	
C14	0.87527 (8)	0.0352 (2)	0.25983 (14)	0.0426 (4)	

### Atomic displacement parameters (Å^2^)

**Table:**

	*U*^11^	*U*^22^	*U*^33^	*U*^12^	*U*^13^	*U*^23^
O1	0.0740 (10)	0.0554 (8)	0.1455 (14)	−0.0116 (7)	0.0756 (10)	0.0060 (8)
C1	0.0571 (11)	0.0449 (10)	0.0648 (11)	−0.0082 (8)	0.0450 (9)	−0.0049 (8)
C2	0.0527 (10)	0.0383 (9)	0.0436 (9)	−0.0017 (7)	0.0331 (8)	−0.0012 (7)
C3	0.0705 (13)	0.0430 (10)	0.0553 (11)	−0.0008 (9)	0.0403 (10)	0.0061 (8)
C4	0.0735 (14)	0.0512 (11)	0.0535 (11)	0.0147 (10)	0.0344 (10)	0.0138 (9)
C5	0.0496 (11)	0.0651 (12)	0.0458 (10)	0.0120 (9)	0.0227 (8)	0.0111 (9)
C6	0.0446 (10)	0.0512 (10)	0.0392 (9)	0.0003 (8)	0.0240 (8)	0.0024 (8)
C7	0.0447 (9)	0.0388 (9)	0.0327 (8)	0.0000 (7)	0.0245 (7)	−0.0022 (7)
C8	0.0394 (9)	0.0327 (8)	0.0406 (9)	−0.0041 (6)	0.0247 (7)	−0.0050 (7)
C9	0.0427 (9)	0.0384 (8)	0.0414 (9)	−0.0003 (7)	0.0276 (7)	−0.0074 (7)
C10	0.0502 (11)	0.0421 (9)	0.0571 (11)	0.0027 (8)	0.0333 (9)	−0.0037 (8)
C11	0.0568 (12)	0.0566 (11)	0.0606 (12)	0.0179 (9)	0.0333 (9)	0.0026 (9)
C12	0.0414 (10)	0.0781 (14)	0.0570 (11)	0.0074 (9)	0.0293 (9)	−0.0030 (10)
C13	0.0464 (10)	0.0645 (12)	0.0558 (11)	−0.0060 (9)	0.0339 (9)	−0.0081 (9)
C14	0.0429 (9)	0.0472 (9)	0.0451 (9)	−0.0033 (7)	0.0303 (8)	−0.0070 (7)

### Geometric parameters (Å, °)

**Table:**

O1—C1	1.2260 (18)	C8—C9	1.500 (2)
C1—C14	1.473 (2)	C8—C8^i^	1.603 (3)
C1—C2	1.475 (2)	C8—H8	0.9800
C2—C3	1.393 (2)	C9—C10	1.392 (2)
C2—C7	1.394 (2)	C9—C14	1.392 (2)
C3—C4	1.372 (2)	C10—C11	1.378 (2)
C3—H3	0.9300	C10—H10	0.9300
C4—C5	1.374 (2)	C11—C12	1.376 (3)
C4—H4	0.9300	C11—H11	0.9300
C5—C6	1.375 (2)	C12—C13	1.367 (2)
C5—H5	0.9300	C12—H12	0.9300
C6—C7	1.388 (2)	C13—C14	1.391 (2)
C6—H6	0.9300	C13—H13	0.9300
C7—C8	1.504 (2)		
			
O1—C1—C14	120.85 (16)	C7—C8—C8^i^	110.23 (10)
O1—C1—C2	121.12 (16)	C9—C8—H8	107.4
C14—C1—C2	117.97 (14)	C7—C8—H8	107.4
C3—C2—C7	119.84 (16)	C8^i^—C8—H8	107.4
C3—C2—C1	118.80 (15)	C10—C9—C14	118.18 (15)
C7—C2—C1	121.31 (14)	C10—C9—C8	119.74 (14)
C4—C3—C2	120.84 (17)	C14—C9—C8	121.98 (13)
C4—C3—H3	119.6	C11—C10—C9	120.78 (16)
C2—C3—H3	119.6	C11—C10—H10	119.6
C3—C4—C5	119.33 (17)	C9—C10—H10	119.6
C3—C4—H4	120.3	C12—C11—C10	120.62 (17)
C5—C4—H4	120.3	C12—C11—H11	119.7
C4—C5—C6	120.59 (17)	C10—C11—H11	119.7
C4—C5—H5	119.7	C13—C12—C11	119.45 (17)
C6—C5—H5	119.7	C13—C12—H12	120.3
C5—C6—C7	121.03 (16)	C11—C12—H12	120.3
C5—C6—H6	119.5	C12—C13—C14	120.73 (17)
C7—C6—H6	119.5	C12—C13—H13	119.6
C6—C7—C2	118.28 (14)	C14—C13—H13	119.6
C6—C7—C8	121.01 (14)	C13—C14—C9	120.23 (15)
C2—C7—C8	120.59 (14)	C13—C14—C1	119.33 (15)
C9—C8—C7	114.57 (13)	C9—C14—C1	120.43 (14)
C9—C8—C8^i^	109.65 (14)		
			
O1—C1—C2—C3	−4.6 (2)	C7—C8—C9—C10	166.38 (13)
C14—C1—C2—C3	172.72 (15)	C8^i^—C8—C9—C10	−69.07 (14)
O1—C1—C2—C7	178.00 (16)	C7—C8—C9—C14	−17.4 (2)
C14—C1—C2—C7	−4.7 (2)	C8^i^—C8—C9—C14	107.14 (13)
C7—C2—C3—C4	0.6 (2)	C14—C9—C10—C11	−0.7 (2)
C1—C2—C3—C4	−176.90 (15)	C8—C9—C10—C11	175.66 (14)
C2—C3—C4—C5	1.7 (3)	C9—C10—C11—C12	0.1 (3)
C3—C4—C5—C6	−1.5 (3)	C10—C11—C12—C13	0.5 (3)
C4—C5—C6—C7	−1.0 (3)	C11—C12—C13—C14	−0.5 (3)
C5—C6—C7—C2	3.2 (2)	C12—C13—C14—C9	−0.1 (2)
C5—C6—C7—C8	−172.91 (15)	C12—C13—C14—C1	−179.91 (16)
C3—C2—C7—C6	−3.0 (2)	C10—C9—C14—C13	0.7 (2)
C1—C2—C7—C6	174.42 (14)	C8—C9—C14—C13	−175.56 (13)
C3—C2—C7—C8	173.14 (14)	C10—C9—C14—C1	−179.51 (14)
C1—C2—C7—C8	−9.5 (2)	C8—C9—C14—C1	4.2 (2)
C6—C7—C8—C9	−164.09 (13)	O1—C1—C14—C13	4.5 (3)
C2—C7—C8—C9	19.91 (19)	C2—C1—C14—C13	−172.84 (14)
C6—C7—C8—C8^i^	71.68 (18)	O1—C1—C14—C9	−175.31 (16)
C2—C7—C8—C8^i^	−104.32 (17)	C2—C1—C14—C9	7.4 (2)

Symmetry codes: (i) −*x*+2, *y*, −*z*+1/2.
